# 1660. Harnessing Handshake Rounds for Infection Prevention: Interventions and Great Catches in the First Year

**DOI:** 10.1093/ofid/ofad500.1493

**Published:** 2023-11-27

**Authors:** Kathleen Martinez, Matthew J Weber, Christine MacBrayne, Sarah K Parker, Ann-Christine Nyquist

**Affiliations:** Children's Hospital Colorado, Arvada, Colorado; University of Colorado/Children's Hospital Colorado, Aurora, Colorado; Children's Hospital Colorado, Arvada, Colorado; University of Colorado/Children's Hospital Colorado, Aurora, Colorado; University of Colorado/Children's Hospital Colorado, Aurora, Colorado

## Abstract

**Background:**

There are national calls to engage Infection Preventionists (IPs) as antimicrobial stewards (AS), but it is unknown how this is translated into action and measurable outcomes. The Association of Professionals in Infection Control and Epidemiology (APIC) and the Society for Healthcare Epidemiology of America (SHEA) 2012 joint position paper states that IPs can benefit ASPs. The paper summarizes key actions for IPs as promoting compliance with standard and transmission-based precautions, care bundle, hand hygiene, and educating staff, patients, and visitors. However, an initial review of the literature did not find any publications outlining IP actions or outcomes as participants in AS.

**Methods:**

In August 2022 an IP was added as available to the existing handshake stewardship rounding team. During rounds the IP observed units for safe infection control practices, asked staff if there were any questions about IC policies or practices, and shared information about circulating illness and COVID-19 policy updates.

**Results:**

Between August 2022 and March 2023, there were 201 interventions. The largest categories included environmental, education, and expired product. 42 (21%) were categorized as a “great catch”, defined as likely to have system-wide impact. Secure chat data was also collected as a baseline to assess change over time.

**Figure:**
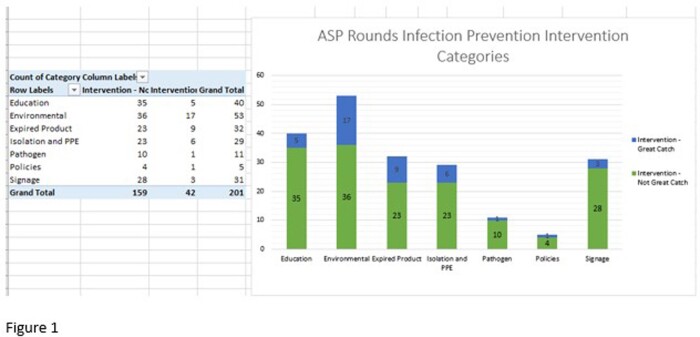

IP interventions during ASP Handshake Rounds

**Figure:**
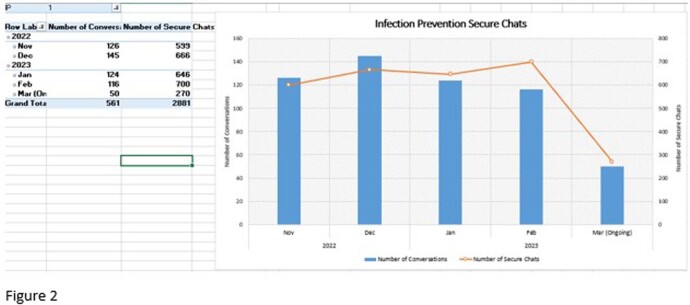

EHR Secure Chats to on call Infection Preventionists

**Conclusion:**

The impact of including an IP during handshake stewardship was great. Impact includes environmental interventions: cleaning and disinfection processes, laundry, waste, damaged structures, construction, and ventilation issues. Educational impact: teaching providers how to access policies, answering questions about isolation and PPE, and the importance of standard precautions and policy changes surrounding COVID-19. Identifying and disposing of expired products and guidance on isolation precautions and PPE. Examples of a great catch included upgrading isolation status, identifying multiple expired products, correcting incorrect PPE or IC in real time, and educating large groups of staff members on critical IC topics. Health care teams and leadership expressed appreciation of education and support provided by the IP during rounds. Integration of IP into Handshake Stewardship will improve IC practices and Joint Commission readiness.

**Disclosures:**

**All Authors**: No reported disclosures

